# Fearful dogs have increased plasma glutamine and γ-glutamyl glutamine

**DOI:** 10.1038/s41598-018-34321-x

**Published:** 2018-10-29

**Authors:** Jenni Puurunen, Katriina Tiira, Katariina Vapalahti, Marko Lehtonen, Kati Hanhineva, Hannes Lohi

**Affiliations:** 1 Department of Veterinary Biosciences, University of Helsinki, Helsinki, Finland; 2 Research Programs Unit, Molecular Neurology, University of Helsinki, Helsinki, Finland; 3 The Folkhälsan Institute of Genetics, Helsinki, Finland; 4 School of Pharmacy, University of Eastern Finland, Kuopio, Finland; 5 Institute of Public Health and Clinical Nutrition, University of Eastern Finland, Kuopio, Finland; 6 LC-MS Metabolomics Center, Biocenter Kuopio, Kuopio, Finland; 7 Department of Equine and Small Animal Medicine, University of Helsinki, Helsinki, Finland

## Abstract

Anxiety-related disorders, including fearfulness are common and leading welfare problems among the worldwide dog population. The etiology of anxieties is complex and affected by genetic and environmental factors. Thus, there is a need for more comprehensive approaches, such as metabolomics, to understand the causes of anxiety and to identify anxiety-related biomarkers for more efficient diagnostic and treatment options. To study metabolic alterations related to canine fearfulness, a non-targeted plasma metabolite profiling was performed in a cohort of 20 fearful and 21 non-fearful dogs. The results showed that nine metabolic features were significantly associated with fearfulness. The most prominent change included increased plasma glutamine and γ-glutamyl glutamine (γ-Glu Gln) in fearful dogs across breeds. Alterations in glutamine metabolism have previously been associated with several psychiatric disorders, indicating the relevance of this finding also in dogs. In addition, we describe a novel breed-specific association between renal biomarker symmetric dimethylarginine (SDMA) and canine fearfulness. These observed metabolic alterations may result from high levels of prolonged psychological stress in fearful dogs.

## Introduction

The estimated worldwide dog population varies from 700,000,000 to one billion dogs, making dog as a man’s best friend^1^. Dog’s behaviour and personality affect not only dog’s but also its owner’s welfare. Especially canine anxiety-related behavioural problems, such as fearfulness, specific phobias and separation anxiety, are common and often severe welfare problems affecting negatively both dog’s and owner’s quality of life^2^. Despite the high prevalence of canine behavioural problems^2–5^, the exact molecular etiology of anxieties still remains largely unknown due to the wide spectrum of environmental and genetic risk factors contributing to the anxiety vulnerability^6–12^. This hinders both diagnostics and treatment plans of canine anxieties.

Similar challenge appears also in humans^13,14^. Since dogs spontaneously manifest analogous behavioural conditions, our best friend may aid human psychiatric research, too^15,16^. Dogs are genetically and physiologically closer to humans when compared to classical animal models, rodents^15,17,18^. Moreover, we share the same environmental and life style factors with dogs. This similarity is reflected in clinical, ethological and pharmacological studies which suggest that the known underlying biochemical mechanisms of anxiety are shared in dogs and humans^15^. However, more comprehensive approaches, such as metabolomics, are needed.

Metabolomics is a comprehensive analysis of small-molecule metabolites (less than 2.0 kDa) present in a biological sample^19,20^. It aims to serve detailed and mechanistic insights into the pathology of diseases by revealing altered metabolic pathways. This may have a particular value in complex pathological states, such as psychiatric disorders^21–25^. As a part of our larger ongoing canine behaviour project, we have previously piloted liquid chromatography combined with mass spectrometry (LC-MS) -based non-targeted metabolite profiling studies of canine fearfulness^26^ and hyperactivity and impulsivity^27^ to understand the feasibility of the approach. Here, our primary goal was to identify fear-specific metabolic alterations in a new diet-controlled study cohort with optimized analytical study conditions.

## Results

### Demographics of the study cohort

The 20 fearful and 21 non-fearful dogs selected to the study were age, sex and breed matched (Table 1, Supplementary Table S1). The non-fearful dogs consisted of 11 Great Danes (7 females and 4 males) and 10 German Shepherds (7 females and 3 males) with no history of fear either towards strangers or in new situations/places, or towards loud noises (human fear, situation fear and fear reaction variables all 0). The age of non-fearful dogs varied from 1.6 to 8.6 years, where the mean age was 4.6 years (median 4.3 years). The fearful dogs (7 female and 4 male Great Danes and 6 female and 3 male German Shepherds) have often (40–100% of occasions) indicated fear towards strangers and/or in new situations. Human fear variable varied from 2 to 30 (mean 15), situation fear variable from 0 to 12 (mean 4.7), and fear reaction variable from 4 to 19.5 (mean 10). The age of the fearful dogs ranged from 1.5 to 8.7 years, where the mean age was 4.7 years (median 4.2 years). A part of the dogs (13 out of 20 case dogs and 11 out of 21 control dogs) was fasting 12 hours before sampling.

**Table 1:** Demographic and behavioural characteristics of study participants.

	Case (N = 20)	Control (N = 21)
Breed (N, German Shepherd/Great Dane)	9/11	10/11
Sex (N, male/female)	7/13	7/14
Age, mean ± SD (years)	4.7 ± 2.3	4.6 ± 2.0
Fasting (N, yes/no)	13/7	11/10
Behavioral test (N, yes/no)	12/8	15/6
Human fear variable, mean ± SD	15.0 ± 7.2	0
Situation fear variable, mean ± SD	4.7 ± 4.3	0
Fear reaction variable, mean ± SD	10.0 ± 4.4	0

SD = standard deviation.

### Plasma metabolite profiling

**Figure 1:**
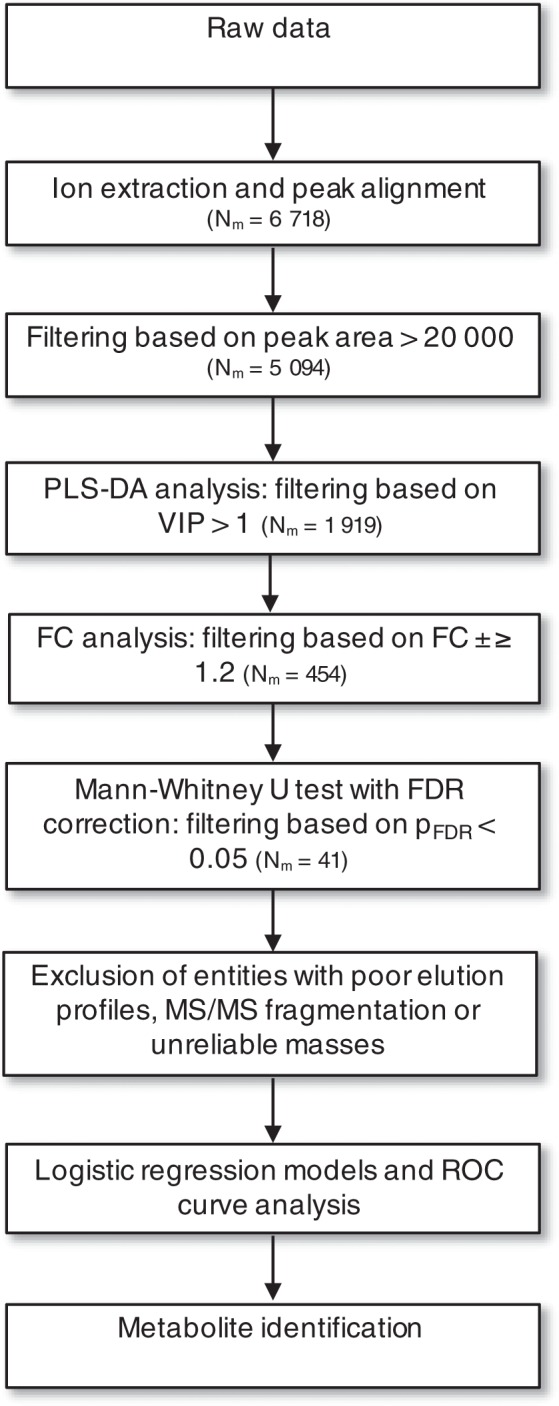
Summary of data processing and statistical analysis steps prior to metabolite identification. N_m_, number of metabolites; PLS-DA, partial least-squares discriminant analysis; VIP, variable importance on projection; FC, fold change.

LC-MS -based metabolite profiling in plasma of fearful and non-fearful dogs resulted in a dataset comprising 6,718 features in four separate analytical runs (1,398 in HILIC ESI(+), 1,381 in HILIC ESI(−), 2,501 in RP ESI(+), and 1,438 in RP ESI(−)). To find out the metabolites explaining most variation between fearful and non-fearful dogs, partial least-squares discriminant analysis (PLS-DA) was applied. Reasonable good separations were observed for models in each analytical approach (RP ESI(+): R^2^X = 0.435, R^2^Y = 0.999, Q^2^ = 0.616; RP ESI(−): R^2^X = 0.435, R^2^Y = 0.997, Q^2^ = 0.679; HILIC ESI(+): R^2^X = 0.557, R^2^Y = 0.999, Q^2^ = 0.208; HILIC ESI(−): R^2^X = 0.416, R^2^Y = 0.999, Q^2^ = 0.594). The variable importance on projection (VIP) values resulting from the PLS-DA models were used to rank the most discriminating metabolites between fearful and non-fearful dogs. After statistical filtering for VIP values > 1, fold change ≥ ±1.2 (positive and negative fold changes indicate higher and lower abundance in fearful dogs, respectively) and Benjamini-Hochberg false discovery rate (FDR) corrected Mann-Whitney U p-values (p_FDR_) < 0.05, 41 metabolic features were left for identification step (Fig. 1). Features with poor chromatographic peak shape or MS/MS fragmentation were excluded. This resulted in a set of five identified metabolites (glutamine, γ-glutamyl glutamine (γ-Glu Gln), symmetric dimethylarginine (SDMA), threo-(Homo)2-isocitrate and 2-oxopimelate) and six unidentified (MW106.0244, MW700.4021, MW134.0194, MW218.0583, MW173.0548, MW100.0162) but statistically significant metabolic features. The differential metabolic features and their characteristics together with identification references are presented in Supplementary Table S2.

### Fearful and non-fearful dogs have differential metabolite profiles

**Figure 2:**
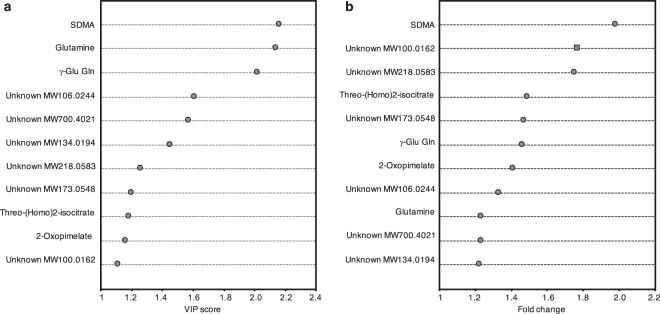
Distributions of VIP scores and fold changes among statistically significant metabolites. (**a**) Distribution of VIP scores among statistically significant metabolites. (**b**) Distribution of fold changes among statistically significant metabolites. Dots indicate positive fold changes whereas squares indicate negative fold changes. SDMA, symmetric dimethylarginine; γ-Glu Gln, γ-glutamylglutamine; VIP, variable importance on projection.

**Figure 3:**
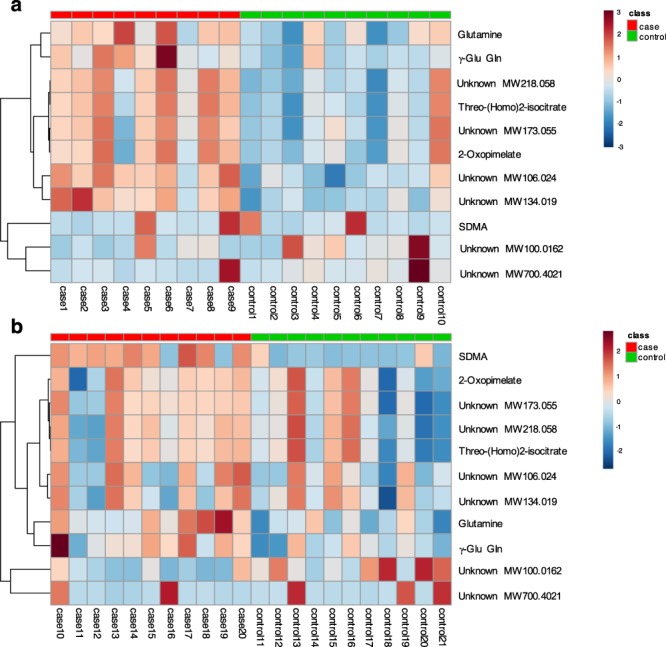
Breed-specific heat maps of metabolic differences between fearful and non-fearful dogs. (**a**) Metabolic differences between fearful and non-fearful German Shepherds. (**b**) Metabolic differences between fearful and non-fearful Great Danes. Heat maps were generated with hierarchical clustering. Included are statistically significant metabolic features having VIP >1, FC ≥ ±1.2 and Mann-Whitney U p_FDR_ < 0.05. The metabolites are listed at the left side of each row, and the subjects are shown at the bottom of each column. The *colour scale* indicates high (*red*) or low *(blue)* metabolite abundance. SDMA, symmetric dimethylarginine; γ-Glu Gln, γ-glutamylglutamine.

A comprehensive set of several statistical analyses showed differences in the metabolite profiles between fearful and non-fearful dogs (Fig. 2, Fig. 3, Supplementary Table S2). According to VIP-values, the most prominent discriminators were SDMA (VIP = 2.16), glutamine (VIP = 2.14) and γ-Glu Gln (VIP = 2.02). In addition, Mann-Whitney U-testing revealed these same metabolites as the most significant ones (glutamine p_FDR_ = 0.005, γ-Glu Gln p_FDR_ = 0.005 and SDMA p_FDR_ = 0.032), and their fold changes indicated also systemic differences between fearful and non-fearful dogs (SDMA fold change 1.98, γ-Glu Gln fold change 1.46 and glutamine fold change 1.23).

### Top metabolites associated with fearfulness

**Figure 4:**
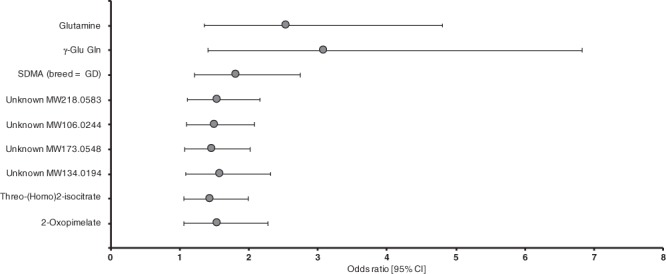
Statistically significant metabolites and their association with fearfulness. Included are metabolites that show statistical significance (p < 0.05) in standard logistic regression models. Error bars indicate 95% confidence limits. γ-Glu Gln, γ-glutamylglutamine; SDMA, symmetric dimethylarginine.

In the logistic regression analyses, breed, sex, age or fasting, as independent variables, did not have significant effects on the phenotype, although, age and breed had significant interactions with unidentified MW100.0162 and SDMA, respectively (Table 2). Glutamine and γ-Glu Gln had the most significant and positive associations with fearfulness (glutamine OR = 2.55 and p = 0.004; γ-Glu Gln OR = 3.10 and p = 0.005) being the most prominent discriminators between fearful and non-fearful dogs (Fig. 4). These metabolites showed also the highest diagnostic ability in ROC curve analysis (γ-Glu Gln AUC = 0.850, glutamine AUC = 0.840) (Table 3). SDMA had a significant interaction with the breed (p = 0.035) in the standard logistic regression analysis and was strongly associated with fearfulness in Great Danes (OR = 1.83 and p = 0.004) but not in German Shepherds (OR = 0.98 and p = 0.902) (Table 2, Fig. 4).

**Table 2:** Results of standard and conditional logistic regression analyses for metabolites that were potentially associated with canine fearfulness according to Mann-Whitney U-test (p_FDR_ < 0.05).

Metabolite	Standard logistic regression						Conditional logistic regression					
	B	SE	p	OR	Lower 95%CI	Higher 95%CI	B	SE	p	OR	Lower 95%CI	Higher 95%CI
Glutamine	0.937	0.322	0.004	2.55	1.357	4.797	1.277	0.638	0.045	3.58	1.026	12.517
γ-Glu Gln	1.133	0.402	0.005	3.10	1.413	6.819	1.887	1.076	0.080	6.60	0.801	54.404
SDMA (breed = GD)^a^	0.602	0.208	0.004	1.83	1.215	2.745	0.340	0.164	0.039	1.41	1.018	1.938
SDMA (breed = GS)	−0.023	0.188	0.902	0.98	0.676	1.412						
Unknown MW218.0583	0.436	0.170	0.011	1.55	1.107	2.159	0.546	0.253	0.031	1.73	1.050	2.834
Unknown MW106.0244	0.417	0.162	0.010	1.52	1.106	2.083	0.292	0.149	0.050	1.34	1.001	1.791
Unknown MW173.0548	0.388	0.162	0.016	1.47	1.074	2.022	0.555	0.246	0.024	1.74	1.075	2.824
Unknown MW134.0194	0.463	0.194	0.017	1.59	1.087	2.322	0.354	0.189	0.061	1.43	0.984	2.064
threo-(Homo)2-isocitrate	0.373	0.162	0.021	1.45	1.057	1.996	0.532	0.260	0.040	1.70	1.024	2.832
2-Oxopimelate	0.440	0.194	0.023	1.55	1.062	2.272	0.607	0.286	0.034	1.84	1.047	3.214
Unknown MW100.0162^b^				0.13	0.023	0.731	−0.367	0.256	0.151	0.69	0.420	1.143
Unknown MW700.4021	−0.107	0.117	0.364	0.90	0.714	1.132	−0.139	0.144	0.334	0.87	0.656	1.154
	Response variable: phenotype (case/control) Explanatory variables: metabolite, breed (Great Dane/German Shepherd), sex (male/female), fasting status (yes/no), age						Response variable: phenotype (case/control) Explanatory variables: metabolite, fasting status (yes/no)					

Metabolic features having Mann-Whitney U FDR-corrected p-values < 0.05 but showing poor chromatographic peak shape and/or MS/MS fragmentation were excluded from logistic regression analyses.

B = coefficient, SE = standard error, OR = odds ratio, CI = confidence interval, γ-Glu Gln = γ-glutamylglutamine, SDMA = symmetric dimethylarginine, GD = Great Dane, GS = German Shepherd

DF = 1

^a^Interaction with breed (p = 0.035) in the standard logistic regression analysis.

^b^Interaction with age (p = 0.045) in the standard logistic regression analysis. OR is presented at the lowest age (18 months). OR multiplies with coefficient e^0.0231^ when age grows with one unit. OR is < 1 at all ages but turns into non-significant at age 86 months or more. Maximum likelihood estimates not shown because of interaction complexity.

**Table 3:** Receiver operator characteristic (ROC) curve analysis of the metabolites.

Metabolite	AUC	Lower 95%CI	Higher 95%CI	Sensitivity, %	Specificity, %	Overall success rate, %
Glutamine	0.840	0.722	0.959	75	71	73
γ-Glu Gln	0.850	0.729	0.971	70	81	76
SDMA^a^	0.827	0.703	0.951	62	79	70
Unknown MW218.0583	0.776	0.619	0.933	75	76	76
Unknown MW106.0244	0.753	0.599	0.906	70	80	75
Unknown MW173.0548	0.745	0.584	0.906	75	76	76
Unknown MW134.0194	0.726	0.568	0.884	70	76	73
threo-(Homo)2-isocitrate	0.742	0.578	0.907	75	75	75
2-Oxopimelate	0.754	0.595	0.914	68	76	72
Unknown MW100.0162^b^	0.812	0.665	0.959	84	67	75
Unknown MW700.4021	0.612	0.437	0.787	55	57	56

AUC = area under the curve, CI = confidence interval, γ-Glu Gln = γ-glutamylglutamine, SDMA = symmetric dimethylarginine.

^a^Interaction with breed.

^b^Interaction with age.

### Glutamine and SDMA together are the best discriminators between fearful and non-fearful dogs

**Figure 5:**
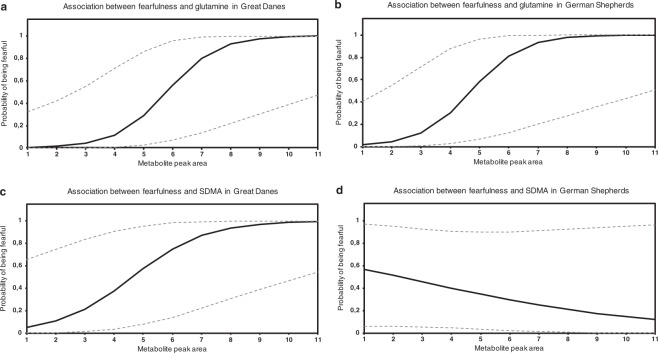
Effects of glutamine and SDMA peak areas on fearfulness in Great Danes and German Shepherds. (**a**) Association between glutamine peak area and fearfulness in Great Danes. The higher the glutamine amount is, the higher is the dog’s probability of being fearful when the dog is Great Dane. (**b**) Association between glutamine peak area and fearfulness in German Shepherds. The higher the glutamine amount is, the higher is the dog’s probability of being fearful when the dog is German Shepherd. (**c**) Association between SDMA peak area and fearfulness in Great Danes. The higher the SDMA amount is, the higher is the dog’s probability of being fearful when the dog is Great Dane. (**d**) Association between SDMA peak area and fearfulness in German Shepherds. SDMA has a slight, non-significant negative association with fearfulness when the dog is German Shepherd. Dashed lines indicate 95% confidence limits. SDMA, symmetric dimethylarginine.

To find the most parsimonious model separating fearful dogs from non-fearful dogs, we performed a multiple logistic regression analysis. High multicollinearity between metabolites (altering the significance of metabolites) hampered the multiple logistic regression analysis but with a stepwise selection, we identified glutamine and SDMA together as the best predictors of fearfulness (Table 4, Fig. 5). However, the significant interaction between SDMA and breed indicated breed-specific association with fearfulness - there was a significant positive association between SDMA and fearfulness in Great Danes but a slight, non-significant negative association between SDMA and fearfulness in German Shepherds (Fig. 5). The ROC curve analysis using variables included in this model produced AUC of 0.930 indicating good specificity and predictive ability for the model (Table 4).

**Table 4:** Results of multiple logistic regression model for the top discriminating metabolites SDMA and glutamine in addition to breed, sex, age and fasting status.

Analysis of maximum likelihood estimates			
Variable	B	SE	p
Intercept	−3.326	2.311	0.150
Breed (GD)	−3.183	1.954	0.103
Sex (female)	−1.085	1.356	0.424
Age	−0.036	0.032	0.255
Fasting (yes)	1.989	1.443	0.168
SDMA	−0.229	0.307	0.456
Glutamine	1.146	0.475	0.016
SDMA*breed (GD)	1.030	0.499	0.039
**Parameter**	**OR**	**Lower 95%CI**	**Higher 95%CI**
SDMA (breed = GD)	2.23	1.181	4.206
SDMA (breed = GS)	0.80	0.436	1.451
Glutamine	3.15	1.240	7.986

B = coefficient, SE = standard error, GD = Great Dane, SDMA = symmetric dimethylarginine. DF = 1. Odds ratio estimates of the significant variables in the model. Odds ratio of SDMA is given in the subgroups of Great Danes and German Shepherd because of the interaction between breed and SDMA.

OR = odds ratio, CI = confidence interval, SDMA = symmetric dimethylarginine, GD = Great Dane, GS = German Shepherd.

Pearson goodness-of-fit statistics: value/DF = 0.781, p = 0.806

Model fit statistics: AIC = 39.651

ROC association statistics: AUC = 0.930, lower 95%CI = 0.850, higher 95%CI = 1.000.

## Discussion

This study characterizes metabolic alterations in canine fearfulness. Fearful dogs showed increased plasma glutamine and γ-Glu Gln levels in addition to breed-specific elevation in plasma SDMA. Although this is not a large-scale study, which is clearly warranted as a follow-up, the potential significance of the findings is emphasized by the fact that glutamine metabolism has been previously linked to anxiety in other species. Additionally, the metabolic changes were observed in two quite different types of breeds, with the exception of SDMA.

Increased plasma glutamine may have various implications since it possesses a wide variety of important functions in the mammal body and its utilization is essential for several organs including the central nervous system (CNS), the intestine, the kidney and the liver^28^. It is a conditionally essential and the most abundant free amino acid in mammals^28,29^. It serves as a precursor for neurotransmitters glutamate and gamma-amino butyric acid (GABA)^30^, antioxidant glutathione^31^, protein, nucleotide and nucleic acid synthesis^28^, a substrate for gluconeogenesis^28^ and ammoniagenesis^32^, and a carrier of nitrogen between organs^33^. Glutamine synthesis can occur in most of the mammalian tissues, but the major organ releasing glutamine into the circulation is skeletal muscle. In turn, the tissues with highest glutamine metabolism are kidney, intestine, immune system and liver^34,35^. Glutamine actions in the brain are mostly mediated by degradation of glutamine to glutamate as a part of glutamate-glutamine cycle^30^. The role of glutamate-glutamine cycle is to maintain normal extracellular glutamate levels in the brain and to remove excess ammonia, formed as a byproduct in the glutamate-glutamine cycle, into circulation^36^. Correct activity and function of this cycle is essential for proper glutaminergic neurotransmission since glutamate is the most abundant and primary excitatory neurotransmitter in the CNS^37^. Indeed, disturbed glutaminergic neurotransmission has been suggested to be involved in a wide spectrum of neuropsychiatric disorders due to the major role of glutamate in fear conditioning^38,39^. Dysfunction of glutamate-glutamine cycle has been proposed in different forms of anxieties^22,23,38,39^, schizophrenia^40–42^, depression^43,44^ and post-traumatic stress disorder^45^.

The plasma glutamine levels can be affected by various factors. Sex and age are known to influence on amino acid plasma concentrations^46^, but the observed difference in glutamine levels between fearful and non-fearful dogs in our study cohort was not affected by these factors. In addition, the amino acid levels may reflect the nutritional status of an individual. However, dogs in our study were fed with the same diet except for one dog and fasting prior blood sampling did not have an effect either. Most of the plasma glutamine originates from skeletal muscle as a result of *de novo* synthesis of glutamine^46^. In addition, high levels of glutamine in the brain may raise its concentration also in plasma, since glutamine is actively cleared to circulation due to the brain’s demand to get rid of excess nitrogen^47,48^. Thus, aberrations in the glutamate-glutamine cycle may be reflected in the peripheral glutamine levels too.

Fearful dogs showed also higher plasma γ-Glu Gln levels. γ-Glu Gln is a γ-glutamyl dipeptide comprised of glutamine joined to the γ-carbon of glutamate. γ-Glutamyl dipeptides are formed as a result of extracellular glutathione breakdown, in a reaction catalyzed by γ-glutamyl transferase (GTT) where the γ-glutamyl moiety released from glutathione binds either water or, in high dipeptide or free amino acid concentrations, another amino acid or dipeptide^49,50^. The biological role of γ-glutamyl dipeptides is unclear, but their formation is highly dependent on antioxidant glutathione metabolism and thus aberrations in glutathione homeostasis may result in altered circulating levels of γ-glutamyl dipeptides^50^. γ-Glu Gln has been characterized in several mammalian tissues^51^ as well as in human plasma^52^ and CSF^52,53^. Aberrations in γ-Glu Gln levels have been observed in the CSF of schizophrenic patients^53^, in the plasma and CSF of hyperammonemic patients^52^, and in the serum of dogs with gallbladder mucocele formation^54^. The increased plasma γ-Glu Gln in fearful dogs may be partially explained by the simultaneous elevation in plasma glutamine abundance. Due to the excess of free glutamine in the circulation, the γ-glutamyl moiety released in the glutathione breakdown favors binding to glutamine over water or other free amino acids or dipeptides and thus more γ-Glu Gln is produced.

Besides increased glutamine and γ-Glu Gln across breeds, a breed-specific elevation in SDMA was observed. A significant and positive association was observed between SDMA and fearfulness in Great Danes, but a non-significant and negative association in German Shepherds. SDMA is a non-proteinogenic amino acid formed by post-translational methylation of both terminal guanidine nitrogens of amino acid arginine^55,56^. It is released via proteolysis into circulation and eliminated mainly by the kidneys. The most well-established biological role of SDMA is indirect inhibition of nitric oxide production. In addition, SDMA may have a role in production of reactive oxygen species. In dogs, SDMA is utilized as a marker of renal function since elevated SDMA levels have been associated with acute kidney injury and chronic kidney disease^57–59^.

Generalized fear reported in the studied dogs may provoke chronic stress with several implications for immune, cardiovascular and neuroendocrine systems and subsequent changes in blood metabolite profiles^60^. Fear stimulus momentarily activates the sympathetic nervous system leading to elevated heart rate, cardiac output and blood pressure in order to prepare the animal to respond to the sudden threat^61^. In addition, cortisol release is stimulated as a result of the hypothalamic-pituitary-adrenal (HPA) axis activation. Highly fearful dogs may suffer from chronic psychological stress resulting in recurrent stimulation of the HPA axis and the sympathetic nervous system with chronic elevations in cortisol as well as catecholamine release with subsequent hypertension^62,63^. Since cortisol regulates *de novo* synthesis of amino acids and degradation of proteins, it may increase amino acid excretion from tissues into circulation^46^. Thus, the observed increase in plasma glutamine in fearful dogs may result from stress-induced changes in cortisol levels. Chronic psychological stress and psychiatric disorders have also been associated with greater levels of oxidative stress^64,65^. Since oxidative stress is known to stimulate glutathione breakdown by activation of GTT^50^, it can result in elevated γ-Glu Gln levels in fearful dogs due to increased release of γ-glutamyl moieties. Interestingly, we found increased levels of oxidative stress promoters, including hypoxanthine and indoxylsulfate, also in our previous pilot metabolomics study of canine fear^26^.

Fear-induced chronic stress may affect also the plasma SDMA levels. Prolonged hypertension is the leading cause of chronic renal failure since elevated blood pressure reduces the glomerular filtration capacity of the kidneys^66,67^. Since circulating SDMA levels are mainly affected by renal factors^59^, our results may suggest that fearfulness can compromise renal function in dogs due to prolonged psychological stress. The owners of fearful dogs did not report their dogs to suffer from any renal diseases during the study, however no clinical examinations by a veterinarian were performed to exclude possible underlying disease states. Plasma SDMA levels are described to increase in very early stages of renal failure^58,59^ and thus the absence of any visible symptoms in fearful dogs is possible. However, elevated SDMA was observed only in fearful Great Danes but not in German Shepherds, and further investigations are needed to clarify the reasons behind this difference.

In this study we observed associations between individual metabolic markers and fearfulness but conducted also multiple logistic regression analysis to identify the best combination of markers with highest discriminating ability between fearful and non-fearful dogs. However, the analysis was hampered by high multicollinearity between metabolites remaining only glutamine and SDMA fitting in the same model. The high multicollinearity is one of largest problems in metabolomics data analysis, since metabolites inherently correlate with each other due to structural and functional similarities between them. Hence, glutamine and SDMA together were the best discriminators that were not multicollinear in our data.

The strengths of this study include a phenotype- and diet-controlled study cohort, fresh plasma as a sample material and the optimization of analytical LC-MS study conditions. However, there are also limitations. Small sample size hinders statistical analyses and warrants a larger replication study to confirm the findings. A challenge lies in the recruitment of highly fearful dogs to the studies since they are more difficult to manage making owners less willing to participate. Therefore, the group of fearful dogs was highly heterogeneous manifesting fearful behaviour from mild to severe.

This study did not identify alterations in the same metabolites as our previous pilot study of canine fearfulness^26^, however, the identified metabolites suggest that the same biological pathways are affected in canine fear. For example, γ-Glu Gln identified in this study and indoxylsulfate and hypoxanthine identified in the earlier study are all related to oxidative stress. There are several obvious reasons preventing the direct replication of results in this study. First, EDTA plasma samples were utilized in this study in comparison to whole EDTA blood samples in the pilot study^26^. The identified metabolite profile is influenced by this change in the sample material. Second, also the sample handling prior to LC-MS analysis was changed since methanol, instead of acetonitrile, was used for metabolite extraction and protein precipitation in the current study. Finally, also dietary factors might have had an effect since in the pilot study the diets of dogs were not controlled^26^ but in the current study the dogs consumed same food two weeks prior to blood sampling. When considering our relatively small sample sizes, it is likely that all these factors have contributed to the discrepancy between these two studies of canine fear.

In summary, we have found differences in the plasma metabolite profiles between fearful and non-fearful dogs, including increased glutamine and γ-Glu Gln in fearful dogs. These results may implicate affected glutamine metabolism in canine fear. Also breed-specific association between renal biomarker SDMA and fearfulness was identified. However, the actual causality between these findings and canine fear remains unsolved - whether the observed changes result from prolonged psychological stress caused by fearfulness or whether they predispose dogs to abnormal fear response. These findings are novel and warrant further validation studies to shed light on the possible role of the identified metabolites in canine fear.

## Methods

### Animals and study design

Twenty fearful and 21 non-fearful Great Danes and German Shepherds were selected from our previously established anxiety study cohort^68^ including a validated owner-filled behavioural questionnaire and a behavioural test for majority of the dogs. The behavioural questionnaire includes both general questions concerning early life experiences, daily routines, diet and exercise, and behaviour-specific questions regarding dog’s reactions in various situations, such as meeting strangers or in novel situations/places. Based on the questionnaire questions concerning dog’s behaviour towards strangers and in new situations, three behavioural variables describing the frequency and intensity of fearful reactions were derived (see questions and their scoring in Supplementary Table S3, and the definitions and calculation of behavioural variables in Table 5). Human fear and situation fear variables describe the frequency and intensity of fearful reactions towards strangers or in new situations, respectively, whereas fear reaction variable describes the frequency and intensity showing fear both towards strangers and in new situations. Dogs that show often (40–100% of occasions) fear towards strangers and/or in new situations were considered as fearful dogs (cases) whereas non-fearful dogs (controls) have never indicated any sign of fear either towards strangers or in new situations/places, or towards loud noises, according to their owners. Behaviour of 12 fearful and 15 non-fearful dogs was verified by a short 5-min validated behavioural test which included three parts: 1) meeting a stranger, 2) exploration in a novel space and 3) novel object test^68^. The tests were conducted by the same trained person.

**Table 5:** The definitions of behavioural variables derived from the canine behavioural questionnaire data.

Variable	Explanation
Human fear variable	Describes the frequency and intensity of fearful reactions towards strangers. Calculated as follows: (sum of fearful behavioural reactions to strangers) × frequency of fear reaction to strangers. Each type of fearful behaviour equaled to 1, except withdrawal which was weighted by multiplying it with 5.
Situation fear variable	Describes the frequency and intensity of fearful reactions in new situations or environments. Calculated as follows: (sum of fearful behavioural reactions in new situations/environments) × frequency of fear reaction in new situation or environment. Each type of fearful behaviour equaled to 1.
Fear reaction variable	Describes the frequency and intensity showing fear towards strangers and in new situations. Calculated as average of human fear score and situation fear score.

Variables describe the dog’s fearful reaction either towards strangers (human fear variable) or in new situations (situation fear variable), and the combination of these reactions (fear reaction variable).

To control the possible effects of diet on the metabolite profiles, all recruited dogs were fed with the same commercial dry food (Royal Canin Maxi Sensible) for two weeks with one week run-in period prior to sampling. All dogs except one (non-fearful dog, German Shepherd, male) consumed the same food. The owners were instructed not to use any other foods or dietary supplements during the two week period and were asked to report of any changes.

To investigate the possible differences in metabolic profiles, EDTA blood samples were collected from each dog by the same trained person followed by immediate isolation of plasma by a portable centrifuge. Plasma samples were kept on ice during shipping and stored in −20 degrees (max 2 months) prior to metabolomics analysis. Most of the samples were taken at the dog’s home and only one dog was sampled at the vet clinic. The sampling time was not standardized to any specific time of the day. The participated dogs were privately owned pet dogs, and informed consent was obtained from the owners. Sample collection was ethically approved by and conducted in accordance with the Animal Ethics Committee of State Provincial Office of Southern Finland (ESAVI/6054/04.10.03/2012) and Royal Canine ethical board (30052016).

### Non-targeted LC-MS metabolite profiling analysis

The sample preparation, instrument parameters and preprocessing of raw data were performed in the LC-MS Metabolomics Center at Biocenter Kuopio (University of Eastern Finland), and they are previously presented in detail^26^. Briefly, an aliquot (100 µl) of plasma samples was mixed with 300 µl of methanol and mixed in vortex at maximum speed 15 s, incubated on ice bath for 15 min, and centrifuged at 16 000 × g for 10 min to collect the supernatant. The supernatant was filtered through 0.2 µm PTFE filters into HPLC vials. From every sample, aliquots of 4 µl were taken and mixed together in one tube and used as the quality control sample in the analysis. The samples were analyzed by the UHPLC-qTOF-MS system (Agilent Technologies, Waldbronn, Karlsruhe, Germany), which consisted of a 1290 LC system, a Jetstream electrospray ionization (ESI) source, and a 6540 UHD accurate-mass quadrupole-time-of-flight (qTOF) mass spectrometry. All samples were analyzed using two different chromatographic techniques, i.e., reversed phase (RP) and hydrophilic interaction chromatography (HILIC). Data were acquired in both ionization polarities; i.e., ESI positive (ESI+) and ESI negative (ESI−). The data acquisition software was MassHunter Acquisition B.04.00 (Agilent Technologies). The quality control samples were injected in the beginning of the analysis and after every 10 samples.

### Data collection

The molecular features were extracted from LC-MS data with MassHunter Qualitative Analysis B.05.00 software (Agilent Technologies) by utilizing “Find by molecular feature” algorithm. Data files (.cef-format) were exported to Mass Profiler Professional software (MPP 2.2, Agilent Technologies) for compound alignment and data preprocessing. In order to reduce noise and remove insignificant metabolic features, only features found in at least 50% of the samples in at least one replicate group (case or control) were included in the analysis.

### Statistical analysis and metabolite identification

The data was exported into Microsoft Excel (2016) and filtered according to peak area >20 000 to exclude small features from further analysis. For multivariate statistical analysis, the pre-processed data from each of the four analytical approaches (RP ESI(+), RP ESI (−), HILIC ESI(+) and HILIC ESI(−)) were subjected to supervised classification algorithm Partial Least-Squares Discriminant Analysis (PLS-DA; Simca-13, Umetrics, Sweden) to find the metabolite features having highest discriminating ability between fearful and non-fearful dogs^69,70^. The data were log10-transformed, pareto-scaled and the model was validated by the Simca-13 internal cross validation, from which cross-validation parameters R^2^X, R^2^Y and Q^2^ were extracted to assess the quality of each PLS-DA model. R^2^X and R^2^Y describe the explained variance of the model whereas Q^2^ indicates the predictive ability of the model^71^. The resulting VIP values for each metabolic feature were integrated in the data.

To focus on the potentially discriminating features associated with fearfulness, only metabolites with VIP >1 and fold change ≥ ±1.2 (positive and negative fold changes indicating higher and lower abundance in case group, respectively) were included in further univariate statistical analyses. Non-parametric Mann-Whitney *U* test was used to analyze differences in metabolic feature distributions between case and control groups, and Benjamini-Hochberg false discovery rate (FDR) correction was used to control for multiple comparisons within each of the four analytical approaches^72^. FDR corrected p-value (p_FDR_) <0.05 was considered as statistically significant. To visualize the differences in metabolite abundances between fearful and non-fearful dogs, heat maps with hierarchical clustering were created in MetaboAnalyst 3.0 (http://www.metaboanalyst.ca/MetaboAnalyst/)^73^.

In order to find the most discriminating metabolic features between cases and controls, we performed multivariable statistical analyses, including conditional and standard logistic regression analyses for each metabolite that remained statistically significant after FDR-controlled Mann-Whitney U-testing. Conditional logistic regression analysis is the conventional method used in pairwise matched case-control studies. However, it can result in greater loss of data than standard logistic regression since observations with missing pair or metabolite value are ignored in conditional analysis^74^. Here, we performed both conditional and standard logistic regression analyses in order to evaluate effect of our pairwise matched study design (the case and control group were age, sex and breed matched). The logistic regression analyses were conducted for each metabolite separately.

In standard logistic regression analyses, the phenotype served as response variable (case/control), and metabolite was the explanatory variable of main interest. Breed (Great Dane/German Shepherd), sex (male/female), fasting status (yes/no) and age were included as potential confounding variables. In conditional logistic regression analyses, the metabolite was the explanatory variable of main interest and non-matched fasting status was included as a potential confounding factor. Interactions between all explanatory variables were studied. To attain reasonable and comparable results in logistic regression analysis, the peak areas of the metabolic features were transformed linearly to scale from 0 to 10. Box-Tidwell test was used to assess the linearity between the continuous predictors and log odds and, if necessary, non-normal distributions of covariates were transformed to normal. In addition, receiver operator characteristic (ROC) curves were calculated to evaluate the specificity and predictive ability of the differential metabolites to be considered as possible biomarkers for fearfulness.

Finally, we conducted multiple logistic regression analysis including the top discriminating metabolic features in the same model in order to reveal the best combination of variables contributing to the differences observed between cases and controls. The analysis was conducted by a stepwise selection. The statistical analyses were performed using SPSS version 22.0.0.1 (IBM Corp.), R project for Statistical Computing version 3.4.1., and SAS sofware version 9.3 (SAS Institute, Cary, NC, USA).

The differential metabolic features were identified, when possible, based on accurate mass and MS/MS fragmentation spectra acquired in the automatic MS/MS analysis during the data acquisition. Online databases, including the Human Metabolome Database, METLIN, MassBank, ChemSpider and KEGG, as well as earlier publications with compound fragmentation patterns were utilized in the identification process^75,76^.

## Electronic supplementary material

Supplementary Dataset 1

## Data Availability

Data is available from the corresponding author on request, as the data is from privately owned pet dogs, and therefore there is a possibility to identify an individual dog from the data.
