# Supplementary material for: Fearful dogs have increased plasma glutamine and γ-glutamyl glutamine
Source: Sci Rep. 2018 Oct 29;8:15976. doi: 10.1038/s41598-018-34321-x (PMC6206014; doi:10.1038/s41598-018-34321-x)
Supplement: Supplementary file 1 — Supplementary Dataset 1 [file 41598_2018_34321_MOESM1_ESM.pdf]

Fearful dogs have increased plasma glutamine and γ-glutamyl glutamine

Jenni Puurunen, Katriina Tiira, Katariina Vapalahti, Marko Lehtonen, Kati Hanhineva & Hannes Lohi

Supplementary table S1. Detailed demographics of study participants. Information includes age, breed, sex, behavioral scores (human fear variable, situation fear variable and fear reaction variable), fasting status and diet of the dog. Dogs numbered from 1 to 20 are fearful dogs (case dogs) whereas dogs numbered from 21 to 41 are non-fearful dogs (control dogs).

	**Age (years)**	**Breed**	**Sex**	**Human fear variable**	**Situation fear variable**	**Fear reaction variable**	**Fasting prior to blood sampling**	**Diet (Royal Canin Maxi Sensible)**
**1**	7.3	German Shepherd	female	9	1	5	yes	yes
**2**	4.2	German Shepherd	female	14	2	8	yes	yes
**3**	3.9	German Shepherd	female	21	6	13.5	yes	yes
**4**	7.5	German Shepherd	female	21	0	10.5	yes	yes
**5**	2.1	German Shepherd	female	12	0	6	yes	yes
**6**	8.2	German Shepherd	female	3	6	4.5	no	yes
**7**	8.7	German Shepherd	male	2	6	4	yes	yes
**8**	4.1	German Shepherd	male	15	4	9.5	yes	yes
**9**	2.9	German Shepherd	male	10	9	8	no	yes
**10**	3.8	Great Dane	female	24	0	12	yes	yes
**11**	2.9	Great Dane	female	21	0	10.5	yes	yes
**12**	2.6	Great Dane	female	10	0	5	yes	yes
**13**	5.0	Great Dane	female	16	10	13	yes	yes
**14**	3.4	Great Dane	female	15	12	15.5	no	yes
**15**	1.6	Great Dane	female	6	0	6	no	yes
**16**	1.5	Great Dane	female	24	9	17	no	yes
**17***	7.5	Great Dane	male	14	12	13	yes	yes
**18**	5.9	Great Dane	male	16	4	10	yes	yes
**19***	6.7	Great Dane	male	30	9	19.5	no	yes
**20**	4.5	Great Dane	male	16	4	10	no	yes
**21**	1.6	German Shepherd	female	0	0	0	yes	yes
**22**	8.2	German Shepherd	female	0	0	0	yes	yes
**23**	5.6	German Shepherd	female	0	0	0	yes	yes
**24**	3.1	German Shepherd	female	0	0	0	yes	yes
**25**	8.6	German Shepherd	female	0	0	0	yes	yes
**26**	5.4	German Shepherd	female	0	0	0	no	yes
**27**	8.2	German Shepherd	female	0	0	0	no	yes
**28**	4.5	German Shepherd	male	0	0	0	yes	yes
**29**	3.6	German Shepherd	male	0	0	0	no	no
**30**	2.8	German Shepherd	male	0	0	0	no	yes
**31**	3.2	Great Dane	female	0	0	0	yes	yes
**32**	1.9	Great Dane	female	0	0	0	yes	yes
**33**	4.3	Great Dane	female	0	0	0	no	yes
**34**	3.0	Great Dane	female	0	0	0	no	yes
**35**	4.6	Great Dane	female	0	0	0	no	yes
**36**	2.7	Great Dane	female	0	0	0	no	yes
**37**	4.1	Great Dane	female	0	0	0	no	yes
**38**	4.3	Great Dane	male	0	0	0	yes	yes
**39**	6.4	Great Dane	male	0	0	0	yes	yes
**40**	5.4	Great Dane	male	0	0	0	yes	yes
**41***	5.4	Great Dane	male	0	0	0	no	yes

* Dog was included also in previous metabolomics study cohort of canine fear (Puurunen, J., Tiira, K., Lehtonen, M., Hanhineva, K. & Lohi, H. Non-targeted metabolite profiling reveals changes in oxidative stress, tryptophan and lipid metabolisms in fearful dogs. *Behav. Brain Funct.* **12,** 7 (2016)).

Supplementary Table S2. Characteristics for the statistically significant metabolites in liquid chromatography-mass spectrometry analysis. Information includes molecular weight (MW), identified ion (*m/z*), retention time (rt), analytical mode and fragment ions in the tandem mass spectrometry (MS/MS fragmentation). Variable importance on projection (VIP) -values, fold change values and Mann-Whitney U FDR-corrected p-values (p_{FDR}) are listed as indicators of statistical significance.

Putative annotation	MW	*m/z*	rt	Mode	MS/MS fragmentation	VIP	Fold change[a]	p_{FDR}[b]
SDMA[1]	202.144	203.151	6.47	HILIC+	ESI(+) 203.150, 172.107, 116.072, 70.067	2.16	1.98	0.032
Glutamine[2]	146.069	147.077	6.17	HILIC+	ESI(+) 147.077, 130.050, 84.045	2.14	1.23	0.005
γ-Glu Gln[3]	275.113	276.119	7.37	HILIC+	ESI(+) 276.193, 242.065, 147.076, 130.049, 57.069	2.02	1.46	0.005
Unknown	106.024	105.017	0.98	HILIC-		1.61	1.33	0.047
Unknown	700.402	701.410	2.97	HILIC+		1.57	1.23	0.032
Unknown	134.019	133.012	0.98	HILIC-		1.45	1.22	0.047
Unknown	218.058	217.051	0.97	HILIC-		1.26	1.75	0.035
Unknown	173.055	172.047	0.97	HILIC-		1.20	1.47	0.036
threo-(Homo)2-isocitrate[4]	220.057	219.050	0.97	HILIC-	ESI(-) 219.054, 173.043, 43.000	1.18	1.49	0.038
2-Oxopimelate[5]	174.051	173.044	0.97	HILIC-	ESI(-) 173.050, 128.041, 43.000	1.16	1.41	0.035
Unknown	100.016	99.009	0.99	HILIC-		1.11	-1.77	0.036

[a] Average fold change when compared against the control group. Fold changes ≥ ± 1.2 were considered as statistically significant. Positive values indicate increased plasma levels in case dogs vs. control dogs, whereas negative values indicate decreased plasma levels in case dogs vs. control dogs

[b] Benjamini-Hochberg false discovery rate (FDR) corrected Mann Whitney U p-value (Benjamini, Y. & Hochberg, Y. Controlling the False Discovery Rate: A Practical and Powerful Approach to Multiple Testing. *Journal of the Royal Statistical Society. Series B (Methodological)* 57**,** 289–300 (1995))

SDMA = symmetric dimethylarginine, γ-Glu Gln = γ-glutamylglutamine

Upperscript numbers denote to identification references as follows: [1] MID44873, Saigusa, D. et al. Determination of Asymmetric Dimethylarginine and Symmetric Dimethylarginine in Biological Samples of Mice Using LC/MS/MS. *Am. J. Anal. Chem.* Vol. 2, pp. 303–313 (2011); [2] MID18, HMDB00641; [3] MT000128, Zhang, J. et al. A metabolomics approach for authentication of Ophiocordyceps sinensis by liquid chromatography coupled with quadrupole time-of-flight mass spectrometry. *Food Res. Int.* Vol. 76, pp. 489–497 (2015); [4] MID71254, KEGG C16597; [5] MID23, KEGG C16588

Supplementary Table S3. Human fear and situation fear related questions in the canine behavioral questionnaire, and the scoring of the answers. Questions concerning fearfulness towards strangers (A), and fearfulness in new situation or environment (B). Human fear variable and situation fear variable were derived from questions A and B, respectively.

**A. Does your dog show shyness or fear when meeting a strange person?**	Score
Withdraws	5
Barks (does not go towards the person)	1
Growls (does not go towards the person)	1
Tail low / between the legs	1
Not willing to make contact	1
Stays close to the owner (even when not under a command)	1
Barks / growls and goes towards a stranger	1
**How often does the dog react fearfully?**	Score
Always, 100% of the times	4
Almost always, 60-100% of the times	3
Often, 40-60% of the times	2
Rarely, 0-40% of the times	1

**B. Does your dog show fear or stress in a new situation or in a new environment?**	Score
Wants out of the situation / new environment	1
Barks	1
Tail low / between the legs	1
Stays still, does not want to explore the new environment	1
Stays close to the owner (even when not under a command)	1
Walks low	1
Pants	1
Trembles	1
**How often does the dog react fearfully?**	Score
Always, 100% of the times	4
Almost always, 60-100% of the times	3
Often, 40-60% of the times	2
Rarely, 0-40% of the times	1
